# Exploring the Interaction of Indole-3-Acetonitrile with Neuroblastoma Cells: Understanding the Connection with the Serotonin and Dopamine Pathways

**DOI:** 10.3390/biomedicines11123325

**Published:** 2023-12-16

**Authors:** Catarina Moura, Ana Salomé Correia, Nuno Vale

**Affiliations:** 1 PerMed Research Group, Center for Health Technology and Services Research (CINTESIS), Rua Doutor Plácido da Costa, 4200-450 Porto, Portugal; cafsm13@gmail.com (C.M.); anncorr07@gmail.com (A.S.C.); 2 CINTESIS@RISE, Faculty of Medicine, University of Porto, Alameda Hernâni Monteiro, 4200-319 Porto, Portugal; 3 ICBAS—School of Medicine and Biomedical Sciences, University of Porto, Rua Jorge Viterbo Ferreira, 228, 4050-313 Porto, Portugal; 4 Department of Community Medicine, Information and Health Decision Sciences (MEDCIDS), Faculty of Medicine, University of Porto, Rua Doutor Plácido da Costa, 4200-450 Porto, Portugal

**Keywords:** indole-3-acetonitrile, serotonin, dopamine, tyrosine, tryptophan, SH-SY5Y cell line, metabolism

## Abstract

Indole-3-acetonitrile, a compound produced by bacteria and plants as a defense and survival signal in response to attacks, has been recently discovered as a metabolite produced by human cancer cells. This discovery suggests a potential association between IAN and cancer progression in patients. Consequently, the aim of this work was to study the effects of IAN on a specific cancer cell line, SH-SY5Y, and elucidate its connection to the serotonin and dopamine pathways by examining the precursors of these neurotransmitters. To achieve this, a cellular viability assay was conducted, along with a morphological evaluation of the cells under both normal and stress conditions. Our results demonstrated that for the highest concentrations in our study, IAN was able to reduce the cellular viability of the cells. Furthermore, when IAN was combined with the amino acids that originate the neurotransmitters, it was possible to observe that in both combinations there was a decrease in the viability of the cells. Thus, IAN may in fact have some influence on both the serotonin and dopamine pathways since changes in cell viability were observed when it was added together with the amino acids. This preliminary study indicates the presence of an interaction between IAN and neuroblastoma cells that justifies further exploration and study.

## 1. Introduction

**Scheme 1:**
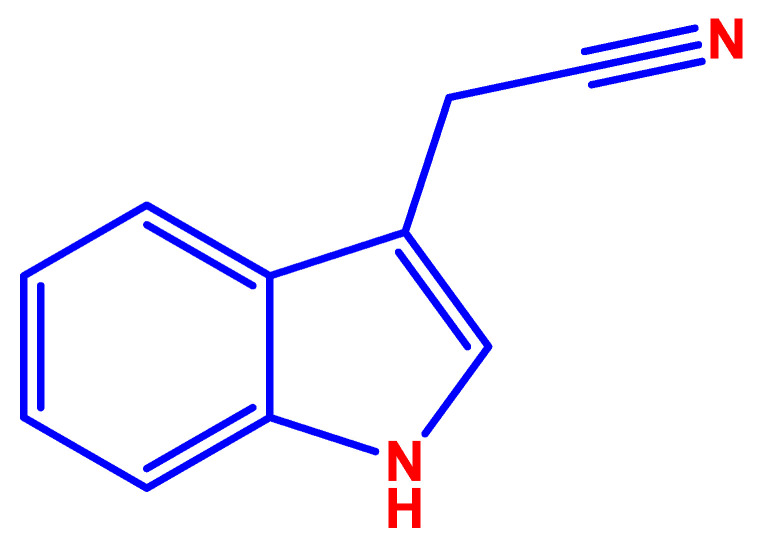
Chemical structure of IAN (indole-3-acetonitrile).

Indole-3-acetonitrile (IAN, Scheme 1) is a member of the auxin indole derivatives family and exerts regulatory control over aspects of plant growth and development. IAN holds significant importance as it serves as a precursor to one of the primary plant hormones, Indole-3-acetic acid. Additionally, in microorganisms, IAN assumes a crucial role in governing growth, development, and interactions with plants [1]. In this way, IAN is produced by bacteria and plants as a defense and survival signal in response to attacks [2]. This compound can be biosynthesized through either tryptophan (Trp)-dependent or Trp-independent pathways, depending on whether Trp is utilized as a precursor [1,3]. The Trp-dependent pathway for IAN biosynthesis starts with the amino acid Trp. The first step involves the conversion of tryptophan to Indole-3-acetaldoxime by the enzyme tryptophan aminotransferase. After that, Indole-3-acetaldoxime is converted to IAN by the enzyme nitrilase [2]. On the other hand, the Trp-independent pathway is less understood compared to the Trp-dependent pathway, which is also controversial. Indeed, the specific enzymes and intermediates involved in this pathway are not fully identified and are subject to ongoing research [4].

In a previous study, IAN was discovered as a part of a new metabolic pathway of Trp in human cancer cells and was considered to be a new metabolite of Trp [5]. IAN was identified as a byproduct generated when breast and melanoma cells underwent stress induced by the introduction of carbidopa. The production of this metabolite emerged as a mechanism employed by these cells to sustain growth and counteract the effects of carbidopa. The observed increase in the viability of breast and melanoma cell lines implies a potential role of IAN in fostering breast cancer and melanoma among Parkinson’s disease patients undergoing treatment with carbidopa drugs [5].

Until now, the presence of IAN had only been found in plants and microorganisms, so the discovery that this metabolite can also be produced by human cancer cells opens up new avenues for research and knowledge. This could confirm that cancer cells are able to establish new mechanisms and new pathways for their growth and development in the human body that are not normally present in the body in normal situations.

Serotonin, a biogenic amino monoamide, is derived from the amino acid Trp [6,7,8]. This monoamide serves as a neurotransmitter in the central nervous system and plays a role as a mediator of motility in the gastrointestinal tract [8]. Recent studies have explored its function in the tumorigenesis of various cancers [9,10]. In this way, the potential stimulatory impact of serotonin on cell proliferation, invasion, dissemination, and tumoral angiogenesis is also being explored. This exploration stems from the hypothesis that this neurotransmitter may be linked to cancer progression, offering a new avenue for potential treatment strategies. Given that both serotonin and IAN derive from Trp, it is interesting to investigate and observe potential connections and links that may exist between these two compounds and their respective pathways.

Dopamine, a brain hormone serving as a neurotransmitter, is synthesized in the brain through a series of steps involving the amino acid tyrosine (Tyr) [11,12,13]. Similar to serotonin, dopamine’s potential association with the progression and development of cancer is acknowledged, although the specifics of this role remain distinct and unclear [14,15].

Hence, serotonin and dopamine share commonalities as neurotransmitters, playing roles in regulating diverse bodily functions. They are both associated with diseases like parkinsonism and depression, and the pathways of these neurotransmitters can also be interconnected and correlated with various types of cancer [9,11,14].

In this study, we aim to examine the behavior of IAN in a distinct cancer cell line, namely the SH-SY5Y neuroblastoma cell line, with a particular focus on neuro-oncology. Additionally, we seek to explore the impact of this compound on the dopamine and serotonin pathways by examining two amino acids that are the precursors of these two neurotransmitters. Additionally, we will also explore how these compounds interact with oxidative stress stimuli by applying hydrogen peroxide to cells, often used in biological studies to induce oxidative stress [16]. Excessive oxidative stress can damage cellular components, implicating it in the pathogenesis of several diseases, including cancer [17].

## 2. Materials and Methods

### 2.1. Materials

Dulbecco’s Modified Eagle’s Medium (DMEM), Fetal Bovine Serum (FBS), and a penicillin–streptomycin mixture were sourced from PAN-Biotech, while thiazolyl blue tetrazolium bromide (MTT; cat. no. M5655), tyrosine (cat. no. T3754), tryptophan (cat. no. T0254), and hydrogen peroxide (30%; Perhydrol^TM^; cat. no. 1.07209) were obtained from Sigma-Aldrich (Merck KGaA, Darmstadt, Germany).

### 2.2. Cell Culture

Human SH-SY5Y neuroblastoma cells (obtained from the American Type Culture Collection, Manassas, VA, USA) were cultured at 37 °C in an environment containing 95% air and 5% CO_2_. The cell culture medium consisted of DMEM supplemented with 10% FBS and 1% penicillin (1000 U/mL)/streptomycin (10 mg/mL). Given that SH-SY5Y cells are adherent, they were maintained in a monolayer and subcultured when reaching a confluence of 75–80%. Before each experiment, trypsin (0.25% trypsin-EDTA) was used to detach the cells. Subsequently, the cells were centrifuged at 1100 rpm for 5 min using a Hettich centrifuge (Tuttlingen, Germany) and then seeded at a density of 4.2 × 10^4^ cells/cm^2^ in 96-well plates for viability assays.

### 2.3. Cell Treatment

Tyr, Trp, and hydrogen peroxide (H_2_O_2_) were dissolved in sterilized water (0.1% in cell culture medium). The amino acids were tested at concentrations of 100 µM, 300 µM, and 500 µM in the cells, and for H_2_O_2_, a stock solution (2 M) was prepared in sterilized water and further diluted in cell culture medium to a working concentration of 132 mM. A 100 mM stock solution of IAN was prepared and dissolved in DMSO (0.1% in cell culture medium). The concentrations of this compound in the cells varied between 0.01 µM and 100 µM. For tyrosine, tryptophan, and IAN/H_2_O_2_ combinations, vehicles were composed of 0.2% DMSO in cell culture medium. For tyrosine and tryptophan/IAN/H_2_O_2_ combinations, the vehicle was composed of 0.3% DMSO in cell culture medium. All the treatments were tested for a period of 24, 48, and 72 h after cell attachment to the plates. For all the combinations tested, both agents were added simultaneously.

### 2.4. Cell Morphology Assessments

After administering the drug treatment, the morphological features of SH-SY5Y cells were examined and recorded utilizing a Leica DMI 6000B Automated Microscope paired with a Leica DFC350 FX camera (Leica Microsystems, Wetzlar, Germany). The cell-containing plate was positioned on the microscope, and the cell images were scrutinized on a computer through the application of Leica Las X imaging software (v3.7.4) (Leica Microsystems, Wetzlar, Germany).

### 2.5. Cell Viability Assays

Cellular viability was assessed at 24, 48, and 72 h after the initiation of cell treatments using MTT assays. Following the removal of the culture medium, 100 µL of MTT solution (0.5 mg/mL in PBS) was introduced into each well. The cells were then shielded from light and incubated for 3 h. Subsequently, the MTT solution was aspirated, and 100 µL of DMSO was added to each well. Finally, absorbance values at 570 nm were measured using an automated microplate reader (Tecan Infinite M200, Zurich, Switzerland).

### 2.6. Statistical and Data Analyses

The results were presented as the mean ± SEM derived from three distinct cell culture preparations. Statistical analyses between control and treatment conditions were conducted through one-way ANOVA tests. Significance was attributed to differences with a *p*-value < 0.05. All statistical analyses and graph constructions were executed using GraphPad Prism 8 software (San Diego, CA, USA).

## 3. Results

### 3.1. Effects of Tyrosine, Tryptophan, and IAN on Cellular Viability

**Figure 1:**
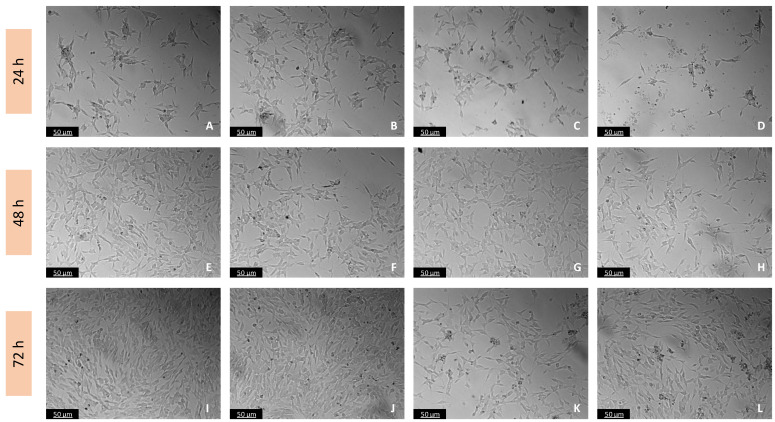
Microscopic visualization of the effects of Tyr on the morphology of SH-SY5Y cells over 24, 48, and 72 h. Cells were treated with (**A**,**E**,**I**) 1% water (vehicle), (**B**,**F**,**J**) 100 µM, (**C**,**G**,**K**) 300 µM, and (**D**,**H**,**L**) 500 µM of Tyr. Representative images were obtained with 100× total magnification from three independent experiments. Scale bar: 50 µM.

**Figure 2:**
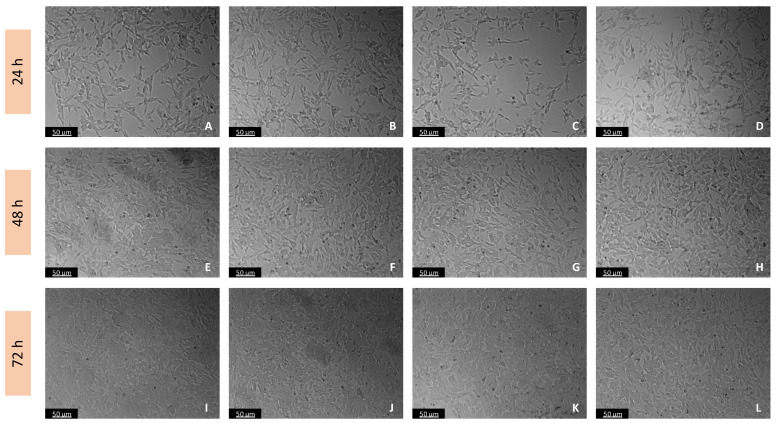
Microscopic visualization of the effects of Trp on the morphology of SH-SY5Y cells over 24, 48, and 72 h. Cells were treated with (**A**,**E**,**I**) 1% water (vehicle), (**B**,**F**,**J**) 100 µM, (**C**,**G**,**K**) 300 µM, and (**D**,**H**,**L**) 500 µM of Trp. Representative images were obtained with 100× total magnification from three independent experiments. Scale bar: 50 µM.

**Figure 3:**
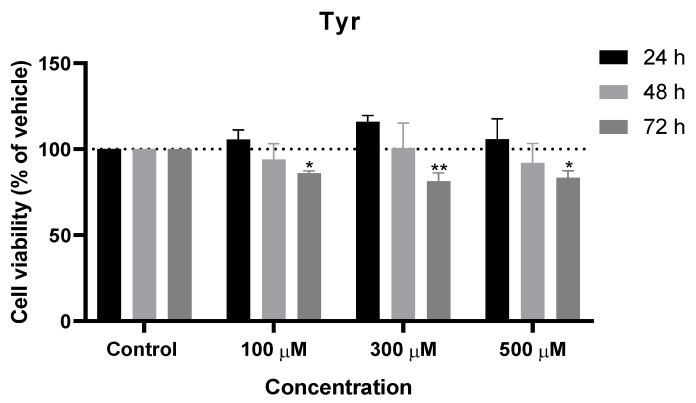
Effect of Tyr on the viability of SH-SY5Y cells. The cells were cultured in the presence of increasing concentrations of Tyr and treated with Tyr for 24, 48, and 72 h. Then, cell viability was determined using the MTT assay. Results are expressed as a percentage of the vehicle-treated control ± SEM of three separate experiments. * Statistically significant vs. control at *p* < 0.05. ** Statistically significant vs. control at *p* < 0.01.

**Figure 4:**
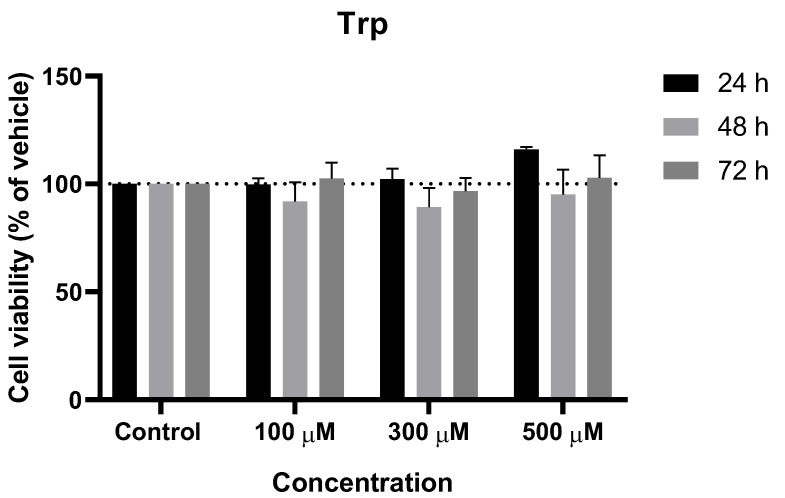
Effect of Trp on the viability of SH-SY5Y cells. The cells were cultured in the presence of increasing concentrations of Trp and treated with Trp for 24, 48, and 72 h. Then, cell viability was determined using the MTT assay. Results are expressed as a percentage of the vehicle-treated control ± SEM of three separate experiments.

To evaluate the effects of Tyr and Trp on the viability of SH-SY5Y cells, the cells were treated with these amino acids at concentrations of 100, 300, and 500 µM over 24, 48, and 72 h of incubation (Figure 1 and Figure 2). Cell viability percentage was evaluated using the MTT assay (Figure 3 and Figure 4).

These results demonstrated that for the 24 and 48 h incubation times studied, both Tyr and Trp showed no significant toxicity toward SH-SY5Y cells, thus not affecting the viability of these cells, either negatively or positively (Figure 3 and Figure 4). However, at 72 h, the three concentrations studied for Tyr showed a significant decrease in the viability of this cell line when compared to the control, but it did not reach viabilities of less than 80%; that is, only a 20% decrease in cell viability was achieved (Figure 3). Thus, for both amino acids tested, no morphological changes were evidenced in the cells (Figure 1 and Figure 2).

**Figure 5:**
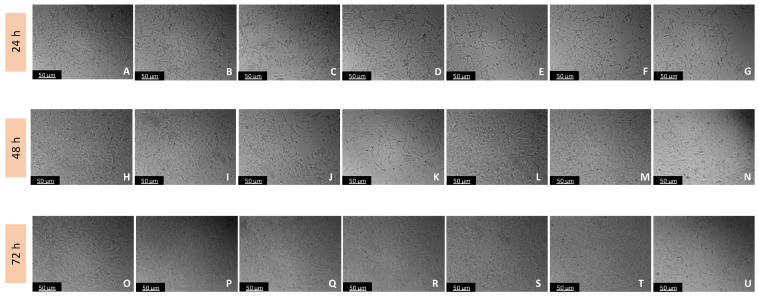
Microscopic visualization of the effects of IAN on the morphology of SH-SY5Y cells over 24, 48, and 72 h. Cells were treated with (**A**,**H**,**O**) 0.1% DMSO (vehicle), (**B**,**I**,**P**) 0.01 µM, (**C**,**J**,**Q**) 0.1 µM, (**D**,**K**,**R**) 1 µM, (**E**,**L**,**S**) 10 µM, (**F**,**M**,**T**) 50 µM, and (**G**,**N**,**U**) 100 µM of IAN. Representative images were obtained with 100× total magnification from three independent experiments. Scale bar: 50 µM.

**Figure 6:**
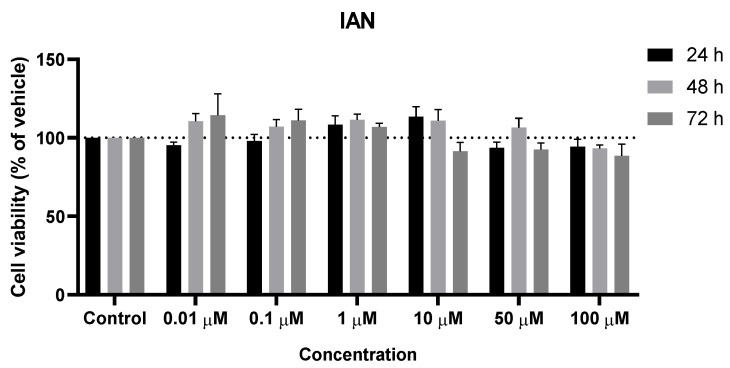
Effect of IAN on the viability of SH-SY5Y cells. The cells were cultured in the presence of increasing concentrations of IAN and treated with IAN for 24, 48, and 72 h. Then, cell viability was determined using the MTT assay. Results are expressed as a percentage of the vehicle-treated control ± SEM of three separate experiments.

After that, to assess the impact of IAN on the viability of SH-SY5Y cells, the cells were exposed to this compound in increasing concentrations over 24, 48, and 72 h (Figure 5). The percentage of cell viability was determined using the MTT assay (Figure 6).

With regard to the study of IAN in SH-SY5Y cells, these results show that this compound did not show any toxicity in this cell line; on the contrary, IAN was able to increase the viability of these cells at concentrations of 0.01, 0.1, 1, and 10 µM at 48 and 72 h, and for 1 µM and 10 µM concentrations at 24 h, an increase in cell viability was also noticed, but not in a statistically significant way when compared to the control group (Figure 6). No notable alterations in cell viability were observed at the other tested concentrations for all the time intervals assessed (Figure 6). Furthermore, there were no observable morphological changes in the cell line under study (Figure 5).

### 3.2. Effects of the Combination of Tyrosine and Tryptophan with IAN on Cellular Viability

**Figure 7:**
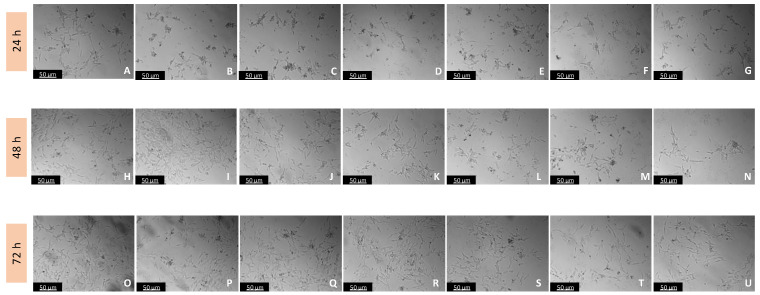
Microscopic visualization of the effects of IAN combined with Tyr on the morphology of SH-SY5Y cells over 24, 48, and 72 h. Cells were treated with (**A**,**H**,**O**) 0.2% DMSO (vehicle), (**B**,**I**,**P**) IAN 0.01 µM + Tyr 500 µM, (**C**,**J**,**Q**) IAN 0.1 µM + Tyr 500 µM, (**D**,**K**,**R**) IAN 1 µM + Tyr 500 µM, (**E**,**L**,**S**) IAN 10 µM + Tyr 500 µM, (**F**,**M**,**T**) IAN 50 µM + Tyr 500 µM, and (**G**,**N**,**U**) IAN 100 µM + Tyr 500 µM. Representative images were obtained with 100× total magnification from three independent experiments. Scale bar: 50 µM.

**Figure 8:**
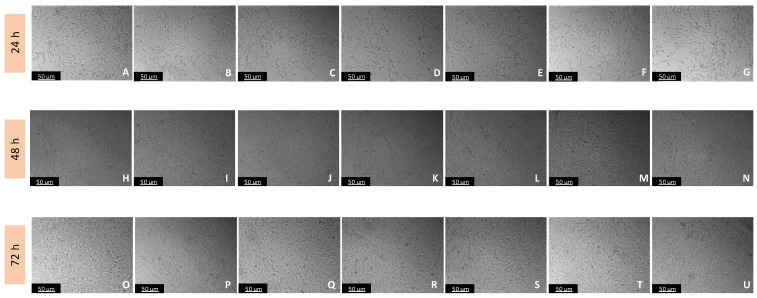
Microscopic visualization of the effects of IAN combined with Trp on the morphology of SH-SY5Y cells over 24, 48, and 72 h. Cells were treated with (**A**,**H**,**O**) 0.2% DMSO (vehicle), (**B**,**I**,**P**) IAN 0.01 µM + Trp 500 µM, (**C**,**J**,**Q**) IAN 0.1 µM + Trp 500 µM, (**D**,**K**,**R**) IAN 1 µM + Trp 500 µM, (**E**,**L**,**S**) IAN 10 µM + Trp 500 µM, (**F**,**M**,**T**) IAN 50 µM + Trp 500 µM, and (**G**,**N**,**U**) IAN 100 µM + Trp 500 µM. Representative images were obtained with 100× total magnification from three independent experiments. Scale bar: 50 µM.

**Figure 9:**
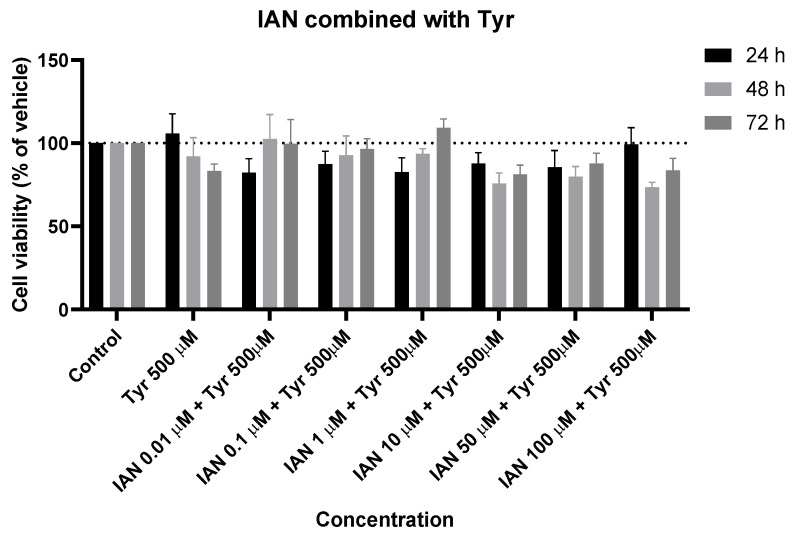
Effect of IAN combined with tyrosine on the viability of SH-SY5Y cells. The cells were cultured in the presence of increasing concentrations of IAN and with a unique concentration of 500 µM of Tyr and treated for 24, 48, and 72 h. Then, cell viability was determined using the MTT assay. Results are expressed as a percentage of the vehicle-treated control ± SEM of three separate experiments.

**Figure 10:**
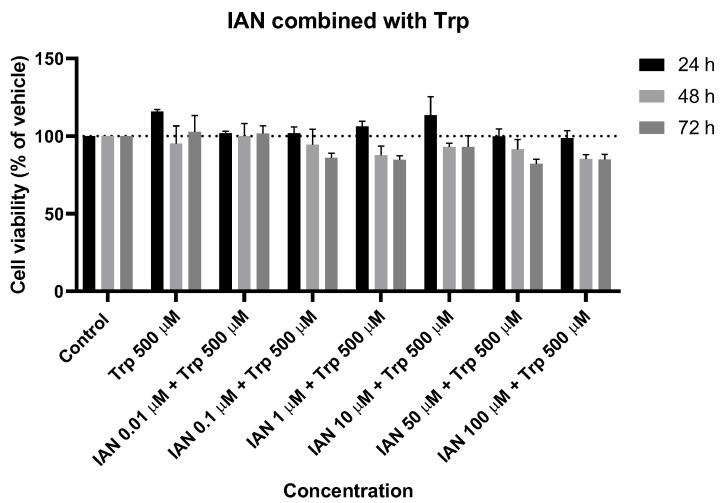
Effect of IAN combined with Trp on the viability of SH-SY5Y cells. The cells were cultured in the presence of increasing concentrations of IAN and with a unique concentration of 500 µM of Trp and treated for 24, 48, and 72 h. Then, cell viability was determined using the MTT assay. Results are expressed as a percentage of the vehicle-treated control ± SEM of three separate experiments.

To assess the impact of combinations involving Tyr and Trp with IAN on SH-SY5Y cell viability, cells were exposed to Tyr and Trp at concentrations of 500 µM, along with increasing concentrations of IAN (0.01, 0.1, 1, 10, 50, and 100 µM). This treatment was carried out over a 24, 48, and 72 h incubation period. Following these periods, morphological analyses were performed on the cells treated with these combinations of compounds (Figure 7 and Figure 8). Subsequently, the MTT assay, as described in the Materials and Methods section, was utilized to determine the percentages of viable cells (Figure 9 and Figure 10).

The results of this study indicate that the two combinations under investigation exhibited a slight decrease in cellular viability (Figure 9 and Figure 10). However, this decrease has not been found to be toxic to cells. Concerning Trp, there was an approximately 15% decrease in cellular viability observed for the studied combinations that achieved a decline in cell viability (Figure 10). In the case of Tyr, a more notable decrease was observed in the combination of IAN 10 µM with Tyr, reaching approximately 76% cell viability (Figure 9). For the other combinations with reduced cell viability, the percentage reached around 18% (Figure 9). This decrease, while not pronounced, had exceptions at certain times and concentrations, in which viability matched the control or showed a slightly higher viability than the control. An example is the combination of IAN 0.1, 1, 10, 50, and 100 µM with Trp 500 µM at 24 h (Figure 10).

### 3.3. Effects of the Combination of Tyrosine, Tryptophan, and IAN with Hydrogen Peroxide on Cellular Viability

**Figure 11:**
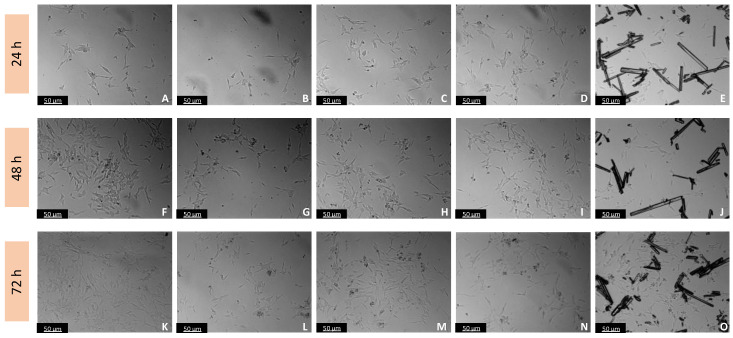
Microscopic visualization of the effects of Tyr combined with H_2_O_2_ on the morphology of SH-SY5Y cells over 24, 48, and 72 h. Cells were treated with (**A**,**F**,**K**) 0.2% DMSO (vehicle), (**B**,**G**,**L**) H_2_O_2_ 132 µM, (**C**,**H**,**M**) Tyr 100 µM + H_2_O_2_ 132 µM, (**D**,**I**,**N**) Tyr 300 µM + H_2_O_2_ 132 µM, and (**E**,**J**,**O**) Tyr 500 µM + H_2_O_2_ 132 µM. Representative images were obtained with 100× total magnification from three independent experiments. Scale bar: 50 µM.

**Figure 12:**
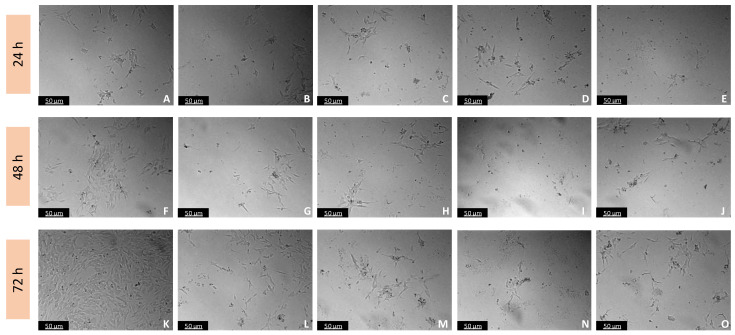
Microscopic visualization of the effects of Trp combined with H_2_O_2_ on the morphology of SH-SY5Y cells over 24, 48, and 72 h. Cells were treated with (**A**,**F**,**K**) 0.2% DMSO (vehicle), (**B**,**G**,**L**) H_2_O_2_ 132 µM, (**C**,**H**,**M**) Tyr 100 µM + H_2_O_2_ 132 µM, (**D**,**I**,**N**) Tyr 300 µM + H_2_O_2_ 132 µM, and (**E**,**J**,**O**) Tyr 500 µM + H_2_O_2_ 132 µM. Representative images were obtained with 100× total magnification from three independent experiments. Scale bar: 50 µM.

**Figure 13:**
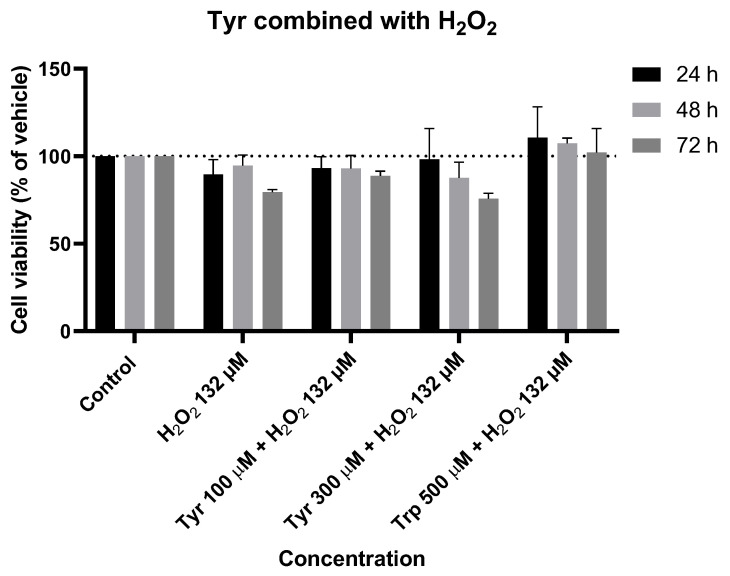
Effect of Tyr combined with H_2_O_2_ on the viability of SH-SY5Y cells. The cells were cultured in the presence of increasing concentrations of IAN, exposed to a constant Trp concentration of 500 µM, and treated for 24, 48, and 72 h. Then, cell viability was determined using the MTT assay. Results are expressed as a percentage of the vehicle-treated control ± SEM of three separate experiments.

**Figure 14:**
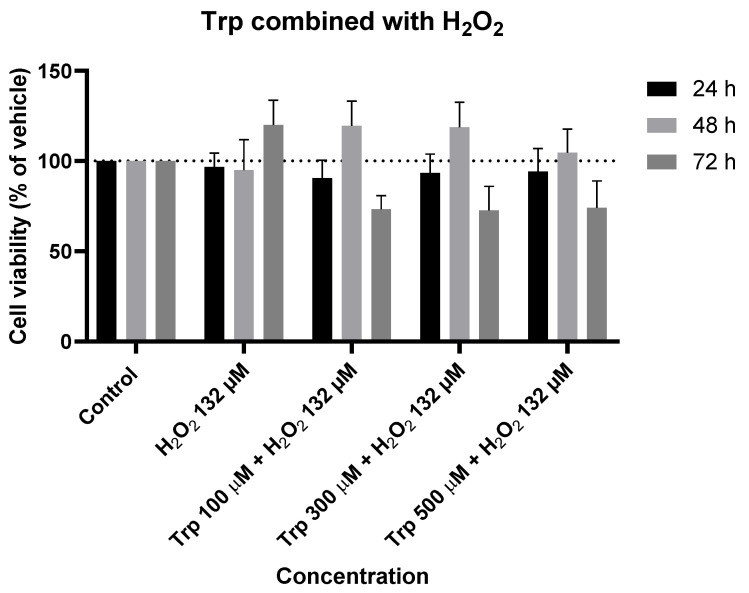
Effect of Trp combined with H_2_O_2_ on the viability of SH-SY5Y cells. The cells were cultured in the presence of increasing concentrations of IAN, exposed to a constant Trp concentration of 500 µM, and treated for 24, 48, and 72 h. Then, cell viability was determined using the MTT assay. Results are expressed as a percentage of the vehicle-treated control ± SEM of three separate experiments.

To evaluate the combinations of Tyr and Trp with H_2_O_2_ on the viability of SH-SY5Y cells, the cells were treated with Tyr and Trp in concentrations of 100, 300, and 500 µM and with 132 µM H_2_O2 (IC_50_ obtained for H_2_O_2_ by the research group for 48 h) [16] for incubation periods of 24, 48, and 72 h. Following this, morphological analyses were performed on the cells treated with this combination of compounds (Figure 11 and Figure 12). The MTT assay was then used to determine the percentage of viable cells (Figure 13 and Figure 14).

These findings indicate that when Tyr is combined with H_2_O_2_, a decrease in cellular viability is noted for concentrations of 100 µM (approximately 90%) and 300 µM (88% viability for 48 h and 76% viability for 72 h) for all the tested durations. However, for the highest concentration studied for this amino acid, a slightly accentuated increase in cell viability is observed (approximately 110% viability). All these results were not statistically significant when compared to the control (Figure 13). Thus, we can conclude that the combination of Tyr and H_2_O_2_ presents some toxicity toward the studied cells, although not statistically significant. Additionally, no alterations in the morphology of the cells were evidenced in this regard (Figure 11). However, the presence of black lines in images E, J, and O (Figure 11) is attributed to the precipitation of Tyr in the medium when added at its maximum concentration.

Regarding Trp combined with H_2_O_2_, the results show that for almost all the concentrations and tested durations, this combination was able to slightly decrease cellular viability in these cells (Figure 14). This suggests that the combination has some toxicity in the cells, as evident in Figure 12, where a slight decrease in the number of cells compared to the control is observed (73% viability for Trp at 72 h vs. 100% viability for the control). This outcome is expected since H_2_O_2_ is known to be toxic to cancer cells [16,18]. At 48 h, all combinations demonstrated a minor increase in cellular viability (an increase of approximately 20% for the 100 and 300 µM concentrations; Figure 14), though not statistically significant. This could imply that during this period, Trp may have inhibited the action of H_2_O_2_ in the cells.

**Figure 15:**
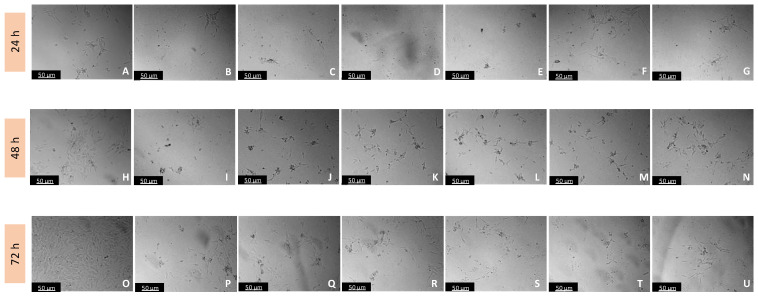
Microscopic visualization of the effects of IAN combined with H_2_O_2_ on the morphology of SH-SY5Y cells over 24, 48, and 72 h. Cells were treated with (**A**,**H**,**O**) 0.2% DMSO (vehicle), (**B**,**I**,**P**) IAN 0.01 µM + H_2_O_2_ 132 µM, (**C**,**J**,**Q**) IAN 0.1 µM + H_2_O_2_ 132 µM, (**D**,**K**,**R**) IAN 1 µM + H_2_O_2_ 132 µM, (**E**,**L**,**S**) IAN 10 µM + H_2_O_2_ 132 µM, (**F**,**M**,**T**) IAN 50 µM + H_2_O_2_ 132 µM, and (**G**,**N**,**U**) IAN 100 µM + H_2_O_2_ 132 µM. Representative images were obtained with 100× total magnification from three independent experiments. Scale bar: 50 µM.

**Figure 16:**
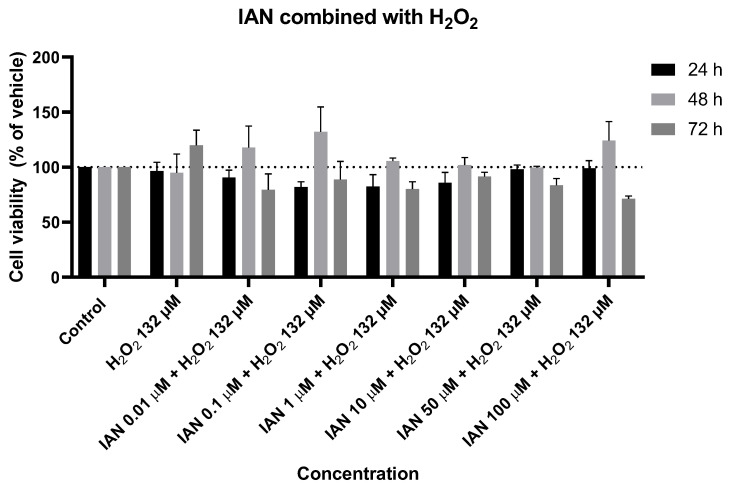
Effect of IAN combined with H_2_O_2_ on the viability of SH-SY5Y cells. The cells were cultured in the presence of increasing concentrations of IAN, exposed to a constant H_2_O_2_ concentration of 132 µM, and treated for 24, 48, and 72 h. Then, cell viability was determined using the MTT assay. Results are expressed as a percentage of the vehicle-treated control ± SEM of three separate experiments.

To assess the combinations of IAN and H_2_O_2_ on the viability of SH-SY5Y cells, the cells were treated with IAN in a concentration range between 0.01 and 100 µM and with 132 µM H_2_O_2_ [16] for incubation periods of 24, 48, and 72 h. Morphological analyses were then performed on the cells treated with this combination of compounds (Figure 15). The MTT assay was then used to determine the percentage of viable cells (Figure 16).

Regarding the study of IAN combined with H_2_O_2_, our results revealed that at 24 and 72 h, and for all the combinations tested, a decrease in cell viability was visualized, even though this decrease was not accentuated except for the combinations of IAN 50 µM and 100 µM with H_2_O_2_ at 24 h (viability of around 99% achieved) (Figure 16). Similar to the combination of Trp and H_2_O_2_, this combination also showed that at 48 h, there was always an increase in the viability of the cells of approximately 20% compared to the control (Figure 16), even though this increase was not statistically significant.

### 3.4. Effects of the Combinations of Tyrosine and Tryptophan with IAN and Hydrogen Peroxide on Cellular Viability

**Figure 17:**
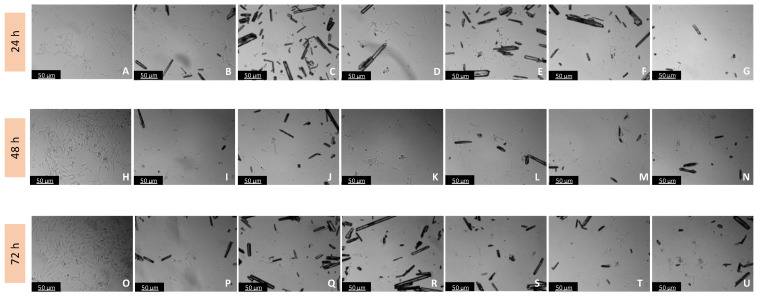
Microscopic visualization of the effects of IAN combined with Tyr and with H_2_O_2_ on the morphology of SH-SY5Y cells over 24, 48, and 72 h. Cells were treated with (**A**,**H**,**O**) 0.3% DMSO (vehicle), (**B**,**I**,**P**) IAN 0.01 µM + Tyr 500 µM + H_2_O_2_ 132 µM, (**C**,**J**,**Q**) IAN 0.1 µM + Tyr 500 µM + H_2_O_2_ 132 µM, (**D**,**K**,**R**) IAN 1 µM + Tyr 500 µM + H_2_O_2_ 132 µM, (**E**,**L**,**S**) IAN 10 µM + Tyr 500 µM + H_2_O_2_ 132 µM, (**F**,**M**,**T**) IAN 50 µM + Tyr 500 µM + H_2_O_2_ 132 µM, and (**G**,**N**,**U**) IAN 100 µM + Tyr 500 µM + H_2_O_2_ 132 µM. The presence of black lines in the images is attributed to the precipitation of Tyr in the medium when added at its maximum concentration. Representative images were obtained with 100× total magnification from three independent experiments. Scale bar: 50 µM.

**Figure 18:**
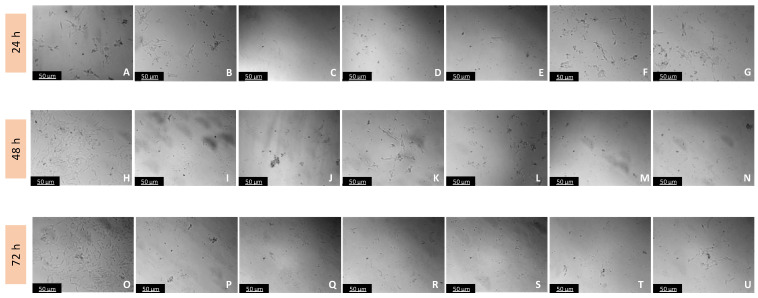
Microscopic visualization of the effects of IAN combined with Trp and with H_2_O_2_ on the morphology of SH-SY5Y cells over 24, 48, and 72 h. Cells were treated with (**A**,**H**,**O**) 0.3% DMSO (vehicle), (**B**,**I**,**P**) IAN 0.01 µM + Trp 500 µM + H_2_O_2_ 132 µM, (**C**,**J**,**Q**) IAN 0.1 µM + Trp 500 µM + H_2_O_2_ 132 µM, (**D**,**K**,**R**) IAN 1 µM + Trp 500 µM + H_2_O_2_ 132 µM, (**E**,**L**,**S**) IAN 10 µM + Trp 500 µM + H_2_O_2_ 132 µM, (**F**,**M**,**T**) IAN 50 µM + Trp 500 µM + H_2_O_2_ 132 µM, and (**G**,**N**,**U**) IAN 100 µM + Trp 500 µM + H_2_O_2_ 132 µM. Representative images were obtained with 100× total magnification from three independent experiments. Scale bar: 50 µM.

**Figure 19:**
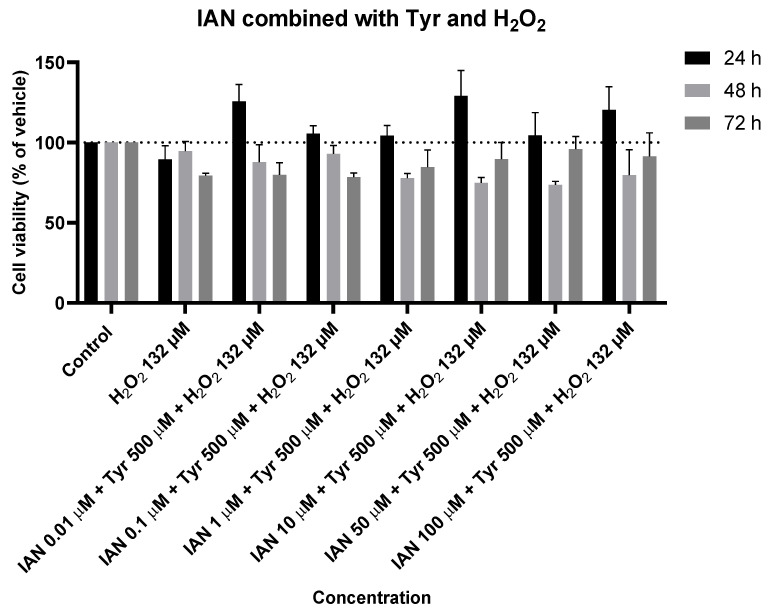
Effect of IAN combined with Tyr and with H_2_O_2_ on the viability of SH-SY5Y cells. The cells were cultured in the presence of increasing concentrations of IAN, maintaining a constant Tyr concentration of 500 µM and a fixed H_2_O_2_ concentration of 132 µM, and treated for 24, 48, and 72 h. Then, cell viability was determined using the MTT assay. Results are expressed as a percentage of the vehicle-treated control ± SEM of three separate experiments.

**Figure 20:**
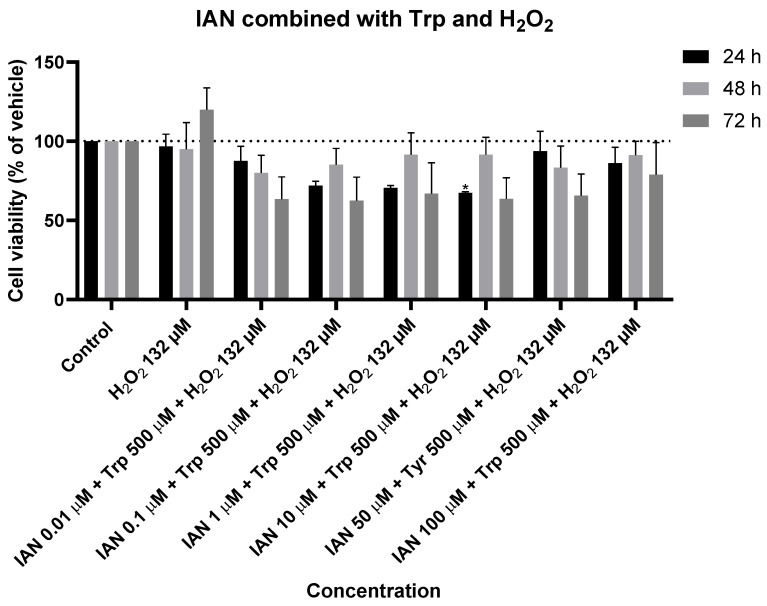
Effect of IAN combined with Trp and with H_2_O_2_ on the viability of SH-SY5Y cells. The cells were cultured in the presence of increasing concentrations of IAN, maintaining a constant Trp concentration of 500 µM and a fixed H_2_O_2_ concentration of 132 µM, and treated for 24, 48, and 72 h. Then, cell viability was determined using the MTT assay. Results are expressed as a percentage of the vehicle-treated control ± SEM of three separate experiments. * Statistically significant vs. control at *p* < 0.05.

To assess the impact of combinations involving Tyr and Trp with IAN and H_2_O_2_ on SH-SY5Y cell viability, the cells were exposed to constant concentrations of 500 µM of Tyr and Trp, increasing concentrations of IAN, and a constant concentration of 132 µM H_2_O_2_ during a 24, 48, and 72 h incubation period. After this, morphological analyses were conducted on the cells treated with this combination of compounds (Figure 17 and Figure 18). Subsequently, the MTT assay was employed to determine the percentage of viable cells (Figure 19 and Figure 20).

Regarding the results of the combinations of Tyr with IAN and with H_2_O_2_, it was possible to observe that, at 24 h, there was a consistent increase in viability, regardless of the concentrations of IAN used, while maintaining a constant concentration of Tyr and H_2_O_2_ (Figure 19). This contradicts our previous findings, in which an increase in viability was only observed at 48 h. At 48 h and 72 h, a decrease in cellular viability is observed (Figure 19), more pronounced at certain concentrations and times. However, this decrease remains statistically insignificant when compared to the control. For instance, at 48 h, the combination of IAN 0.01 µM + Tyr 500 µM + H_2_O_2_ 132 µM resulted in a cell viability of 78%, indicating a decrease of approximately 22%. Similarly, at 72 h, the combination of IAN 50 µM + Tyr 500 µM + H_2_O_2_ 132 µM resulted in a cell viability of 73%, representing a decrease of around 27%. These results demonstrate that this combination of compounds is not toxic to SH-SY5Y cells.

In relation to the outcomes of the combination of Trp with IAN and with H_2_O_2_, it is observed that, for all combinations and throughout the study periods, there was a reduction in cell viability (Figure 20). This indicates that these combinations do indeed exhibit some toxicity toward the cells under investigation. Notably, a statistically significant decrease in cell viability was achieved at 24 h with the combination of IAN 10 µM + Trp 500 µM + H_2_O_2_ 132 µM, reaching a viability of 67% compared to the control (Figure 20). These findings are further supported by Figure 18, where a decrease in the number of cells is evident for all combinations when compared to the control figure.

As shown in Table 1, there was nearly always a slight decrease in cell viability at 24 and 72 h under all of the conditions that were evaluated. However, it is also possible to see that for the highest concentration of tryptophan under study, there was an increase in cell viability both at 24 h and 72 h. The period in which the greatest increase in viability was seen was at 24 h, with the cells under study achieving a viability percentage of approximately 116%. On the other hand, when Trp was combined with H_2_O_2_ and IAN, the tendency for all combinations was always a slight decrease in cell viability, which was barely noticeable at 24 h but a little more evident at 72 h. Regarding IAN, the table also illustrates that, for the highest concentration of this compound under study, there is a tendency for a decrease in the percentage of viable cells, although not as pronounced as in the results mentioned above. Therefore, it is possible to emphasize that the combination of IAN and H_2_O_2_ causes the biggest drop in cell viability under these circumstances, with a 28% reduction.

In Table 2, we observe a consistent decrease in cellular viability over time for nearly all the studied conditions compared to the control group, as occurred in Table 1. At 24 h, an increase in cell percentage is evident in three conditions: exposure to Tyr alone, Tyr combined with H_2_O_2_, and Tyr combined with IAN and H_2_O_2_. However, by 72 h, a decrease in cell viability is observed for these concentrations. Specifically, when IAN is added to the cells alone, a slight decrease in cell percentage is visualized, and this reduced trend aligns with time, resulting in reduced cell viability from 24 to 72 h. The most significant reductions in cell viability are noted in two distinct conditions: when IAN is combined with H_2_O_2_ and when Tyr is combined with IAN and H_2_O_2_, showing decreases of approximately 28% and 29%, respectively.

## 4. Discussion

Since many pathways naturally mutate in cancer patients, playing a significant role in the advancement of the disease, it is crucial to try to discover as many mutated pathways as possible in this disease, regardless of whether they are genetic, epigenetic, or even neuronal pathways. As scientists already know, the Trp pathway is also one of the pathways that is frequently mutated in several cancers [5]. Therefore, it is also important to investigate the compounds derived from this amino acid pathway, including serotonin. Indeed, the serotonergic signaling system is frequently dysregulated in several types of cancer, affecting cancer growth in different ways depending on its concentration and the serotonergic receptors involved [19]. Furthermore, Tyr plays a pivotal role in oncology, as highlighted earlier in the introduction. Its involvement in key cellular processes with implications for cancer progression underscores the need for exploration at the cellular/molecular level.

IAN, a compound originating from the Trp pathway, has been recently discovered and synthesized by breast and melanoma tumor cells when exposed to carbidopa, a drug used to treat Parkinson’s disease [5]. This discovery led to research into this compound in another cell line, SH-SY5Y, and its impact on two neuronal pathways, the serotonin and dopamine pathways.

Thus, in this study, we explored the effects of IAN on SH-SY5Y neuroblastoma cells, specifically focusing on the impact of IAN itself, Trp, and Tyr. Our findings presented a notable divergence from previous studies conducted on melanoma and breast cancer cells. In those studies, IAN was observed to enhance cell viability, but in this research, we found that higher concentrations of IAN reduced the viability of SH-SY5Y cells. This indicates a unique response of these neuroblastoma cells to IAN, differing significantly from the response observed in melanoma and breast cells. These findings highlight the significance of cell-specific characteristics and metabolic pathways in drug responses. SH-SY5Y cells, with their distinct neuronal origins and metabolic profiles, react differently to IAN compared to melanoma and breast cancer cells, which also have their own unique cellular structures/functions. To fully understand these responses, comparative studies are necessary, focusing on how IAN interacts with each cell type’s signaling pathways and metabolic processes. Nevertheless, this exploratory research emphasized the importance of tailoring research to specific cell types that represent different types of cancer.

Furthermore, when assessing the impact of the amino acids Trp and Tyr, our results showed a more pronounced decrease in cell viability in response to Tyr exposure. This suggests that Tyr has a more substantial cytotoxic effect on SH-SY5Y cells compared to Trp. The observed difference in cytotoxic effects between Trp and Tyr on SH-SY5Y cells could be attributed to several factors. A possible explanation might be related to oxidative stress. In fact, Tyr can generate tyrosyl radicals through oxidation, potentially causing cellular oxidative damage. In contrast, Trp may produce fewer reactive species, resulting in reduced oxidative stress and cytotoxicity [20,21]. Also, Tyr and Trp follow distinct cellular pathways. Tyr serves as a precursor for catecholamines like dopamine, norepinephrine, and epinephrine [22], with excess accumulation potentially causing oxidative stress and cytotoxicity. In contrast, Trp undergoes metabolism through the kynurenine and serotonin pathways, which are involved in mood regulation and stress response [23]. Indeed, these differential pathways may contribute to the different effects.

Moreover, in our evaluation of the combined effects of H_2_O_2_ with IAN, Tyr, or Trp, our results showed a more pronounced reduction in cell viability than when tested without the combination. This outcome aligns with expectations, given that this compound acts as a stressor in tumor cells. Notably, at the elevated concentration of Tyr investigated, our findings revealed a contrary effect: a slight increase in cell viability. This apparent contradiction may be elucidated by considering that the stress-inducing effects of both compounds, when combined, could potentially counterbalance each other, leading to the observed increase in cell viability.

Additionally, when the combined effects of IAN with Trp or Tyr were explored, we observed a decrease in cell viability in both cases. However, this decrease was not marked. This outcome highlights the complex interplay between these metabolites and their combined effects on neuroblastoma cell viability.

Thus, this study contributes valuable insights into the differential responses of SH-SY5Y cells to this metabolite compared to other cancer cell types previously explored and underscores the importance of context-specific investigations in cancer research.

## 5. Conclusions

This study accentuates the critical role of cell-specific attributes and metabolic pathways in determining the response to therapeutic interventions and shaping drug responses. In SH-SY5Y neuroblastoma cells, IAN demonstrated a distinctive response, causing a reduction in cell viability at higher concentrations, in contrast to its enhancing effect observed in melanoma and breast cells. Notably, Tyr exhibited a more pronounced cytotoxic impact on SH-SY5Y cells compared to Trp, potentially attributed to oxidative stress and distinct cellular pathways. This cytotoxic effect was further pronounced when combining H_2_O_2_ with IAN, Tyr, or Trp, resulting in a significant decrease in cell viability, with a slight elevation observed at elevated Tyr concentrations. The complex interplay between these metabolites and their combined effects on neuroblastoma cell viability highlights the critical need for context-specific investigations in cancer research.

## Figures and Tables

**Table 1:** Percentage of SH-SY5Y cells at different conditions using Trp, IAN, combinations of both, and combinations of both with H_2_O_2_ evaluated at 24 and 72 h.

Conditions	% of Cells at 24 h	% of Cells at 72 h
Control	100	100
Trp 500 µM	116	103
IAN 100 µM	94	88
Trp 500 µM + IAN 100 µM	99	85
Trp 500 µM + H_2_O_2_ 132 µM	94	74
IAN 100 µM + H_2_O_2_ 132 µM	99	71
Trp 500 µM + IAN 100 µM + H_2_O_2_ 132 µM	86	79

**Table 2:** Percentage of SH-SY5Y cells at different conditions using Tyr, IAN, combinations of both, and combinations of both with H_2_O_2_ evaluated at 24 and 72 h.

Conditions	% of Cells at 24 h	% of Cells at 72 h
Control	100	100
Tyr 500 µM	106	83
IAN 100 µM	94	88
Tyr 500 µM + IAN 100 µM	99	84
Tyr 500 µM + H_2_O_2_ 132 µM	111	102
IAN 100 µM + H_2_O_2_ 132 µM	99	71
Tyr 500 µM + IAN 100 µM + H_2_O_2_ 132 µM	120	91

## Data Availability

Data are contained within the article.
